# Potential significance of uncovered self‐expandable metal stents for distal malignant biliary obstruction: A propensity score‐adjusted competing risk regression analysis

**DOI:** 10.1002/deo2.166

**Published:** 2022-09-20

**Authors:** Yuichi Torisu, Masafumi Chiba, Masayuki Kato, Yuji Kinoshita, Takafumi Akasu, Tomoya Kanai, Yoichi Tomita, Nana Shimamoto, Takahiro Abe, Keisuke Kanazawa, Shintaro Tsukinaga, Masanori Nakano, Chisato Saeki, Kazuki Sumiyama, Masayuki Saruta

**Affiliations:** ^1^ Department of Internal Medicine Division of Gastroenterology and Hepatology, The Jikei University School of Medicine Tokyo Japan; ^2^ Department of Endoscopy The Jikei University School of Medicine Tokyo Japan; ^3^ Department of Internal Medicine Division of Gastroenterology, Fuji City General Hospital Shizuoka Japan

**Keywords:** covered self‐expandable metal stent, distal malignant biliary obstruction, proper use, time to recurrent biliary obstruction, uncovered self‐expandable metal stent

## Abstract

**Objectives:**

Selection criteria for self‐expandable metal stents (SEMSs) with or without cover during palliative treatment of distal malignant biliary obstruction (DMBO) remain unclear. We evaluated factors associated with time to recurrent biliary obstruction (TRBO) in fully covered SEMSs (FCSEMSs) and uncovered SEMSs (UCSEMSs).

**Methods:**

We retrospectively analyzed consecutive patients with DMBO who received a SEMS. TRBO was determined using the Kaplan–Meier analysis, and complications were compared between the FCSEMS and UCSEMS groups. After TRBO‐associated factors were extracted using multivariate competing‐risks regression (CRR), propensity score‐adjusted CRRs were performed to verify their robustness.

**Results:**

There were 180 patients (66 FCSEMSs and 114 UCSEMSs) enrolled in this study. There was no significant difference between median TRBO in the FCSEMS and UCSEMS groups (275 vs. 255 days, *p* = 0.67). Complications were more frequent in the FCSEMS than UCSEMS group (21.2% vs. 8.8%; *p* = 0.023). Multivariate CRR for TRBO‐associated factors revealed that “pancreatic ductal carcinoma (PDAC) treated with UCSEMS” was the only independent predictor of TRBO (*p* = 0.03). Similarly, the propensity score‐adjusted CRRs showed no significant difference in TRBO in “FCSEMS” vs “UCSEMS” (*p* = 0.96); however, there was a significant difference in “PDAC using UCSEMS” vs “other” (*p* = 0.043). In the palliative care group including any DMBO without chemotherapy, the first quartile of the TRBO of UCSEMS was 100 days.

**Conclusions:**

UCSEMSs are a possible option for both patients with DMBO arising from PDAC and for patients with any DMBO receiving palliative care who should avoid SEMS‐related complications.

## INTRODUCTION

Endoscopic stent insertion is an established means of palliating distal malignant biliary obstructions (DMBOs).^1^, ^2^ Compared to plastic stents (PSs), self‐expandable metal stents (SEMSs) are widely used and associated with time to recurrent biliary obstruction (TRBO), lower risk of stent dysfunction/cholangitis, and fewer reinterventions.^3^, ^4^ However, selecting fully covered SEMSs (FCSEMs) and uncovered SEMSs (UCSEMs) is difficult. Over 11 randomized controlled trials that compared the two, FCSEMSs were superior in four^5^, ^6^, ^7^, ^8^ and UCSEMSs in two.^9^, ^10^ Further, no significant difference in the cumulative patency time was observed over five randomized controlled trials.^11^, ^12^, ^13^, ^14^, ^15^ Moreover, there were no significant differences found in stent dysfunction, overall complications, or patient survival over eight meta‐analyses,^16^, ^17^, ^18^, ^19^, ^20^, ^21^ with the exception of stent dysfunction in three meta‐analyses.^22^, ^23^, ^24^ Therefore, there is no consensus on SEMS type for palliation of DMBO. Despite no significant difference in the cumulative patency time between the two, FCSEMs are preferred over UCSEMSs due to their ease of removal and replacement.^25^

While the use of FCSEMSs for DMBO is becoming more common^24^; however, the criteria governing their selection remain unclear in patients ineligible for surgery and seeking palliation or chemotherapy. Therefore, we investigated factors associated with recurrent biliary obstruction (RBO) and, in this work, propose criteria for selecting FCSEMS or UCSEMS based on patient‐specific factors.

## METHODS

### Patients

**FIGURE 1:**
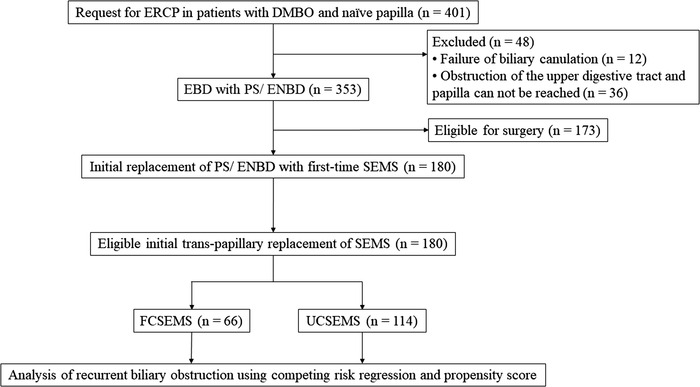
Patients’ flow. DMBO, distal malignant biliary obstruction; EBD, endoscopic biliary drainage; ENBD, endoscopic nasobiliary drainage; ERCP, endoscopic retrograde cholangiopancreatography; FCSEMS, fully covered self‐expandable metallic stent; SEMS, self‐expandable metallic stent; UCSEMS, uncovered self‐expandable metallic stent

The present retrospective cohort study included consecutive patients with DMBO who were not eligible for surgery, thereby electing patients undergoing chemotherapy or terminal care. All patients underwent initial trans‐papillary SEMS placement as a second session of endoscopic retrograde cholangiopancreatography (ERCP) after endoscopic biliary drainage with a PS or endoscopic nasobiliary drainage comprising the first session between January 2012 and December 2020 at The Jikei University Hospital and Fuji City General Hospital, Japan (Figure 1). The diagnosis of malignancy was based on pathological and/or typical radiological findings. DMBO was confirmed in all patients using laboratory imaging data, including ERCP, abdominal enhanced computed tomography, and magnetic resonance cholangiopancreatography. The study was approved by the Ethics Committee of Jikei University School of Medicine [No. 31‐023(9522)] and complied with all standards established by the Declaration of Helsinki. Written informed consent to undergo ERCP was obtained from all patients prior to participation. The opportunity to opt‐out of this study participation was also provided, and the requirement for informed consent was waived as this was an anonymous retrospective observational study (opt‐out method of informed consent).

### Procedures

**FIGURE 2:**
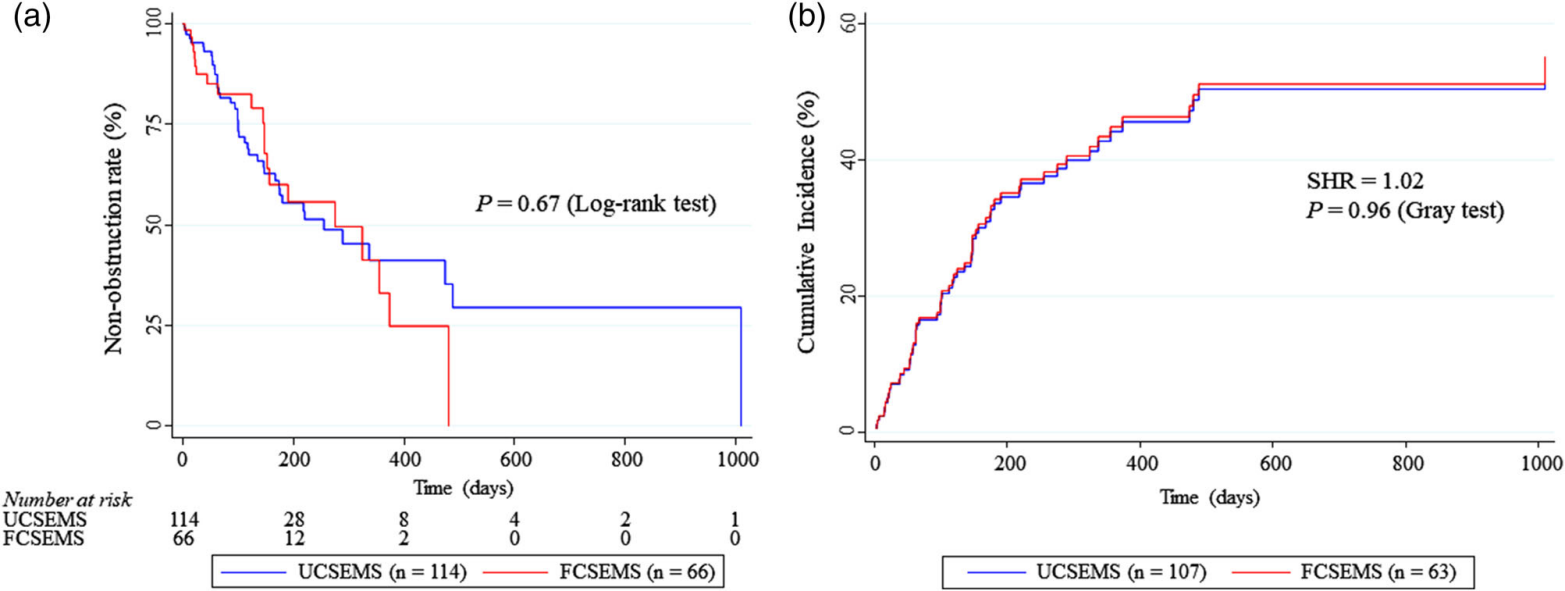
Comparison of time to recurrent biliary obstruction between fully covered and uncovered self‐expandable metal stent groups. (a) Kaplan–Meier curves of time to recurrent biliary obstruction. In the Kaplan–Meier analysis, patients who died were censored. (b) Time to recurrent biliary obstruction was determined using propensity score‐adjusted competing‐risks regression. In the competing risk model for recurrent biliary obstruction rates, mortality was assigned as the competing risk. All variables listed in Table 1 were adjusted using propensity score analysis.

ERCP was performed under fluoroscopy by experts who had performed at least 200 ERCPs per year, or by trainees with expert supervision, depending on the situation. All patients who underwent ERCP were conscious but sedated with intravenous flunitrazepam and pethidine during the procedure. Initial placement of a SEMS was performed in the following patients: 1) patients with DMBO and naïve papilla who underwent an initial endoscopic biliary drainage using PS or endoscopic nasobiliary drainage (Figure 1), 2) patients with malignant pathological diagnosis based on the results of ERCP or endoscopic ultrasound‐guided fine needle aspiration, and 3) inoperable patients. We also routinely perform endoscopic sphincterotomy before FCSEMS insertion but not before UCSEMS insertion, because UCSEMS itself empirically does not seem to block the pancreatic duct opening. Each SEMS was placed across the duodenal papilla with approximately 1 cm of the distal end of the stent protruding into the duodenal lumen. The SEMS length was based on each patient's individual anatomy and stricture length. The decision of the type of SEMS (FCSEMS/UCSEMS), stent manufacturer, and diameter of SEMS (10/8/6 mm) was determined at the physician's discretion.

### Definitions

Based on “the TOKYO criteria 2014,” RBO was defined as a composite endpoint of either symptomatic occlusion or symptomatic migration; TRBO was defined as the time from SEMS placement to the RBO.^26^ All patients were followed up until June 30, 2021, by right censoring.^27^ Patients were censored if they were lost to follow‐up without RBO. The definitions for technical and functional success and nonRBO adverse events were based on TOKYO 2014 criteria.

### Statistics

When appropriate, data are presented as means (±standard deviations), medians (interquartile range), or frequencies. Fisher's exact test and the Chi‐square test were used to compare categorical variables; the Mann–Whitney *U*‐test was used to compare continuous variables. Missing values were excluded for complete case analysis.

**FIGURE 3:**
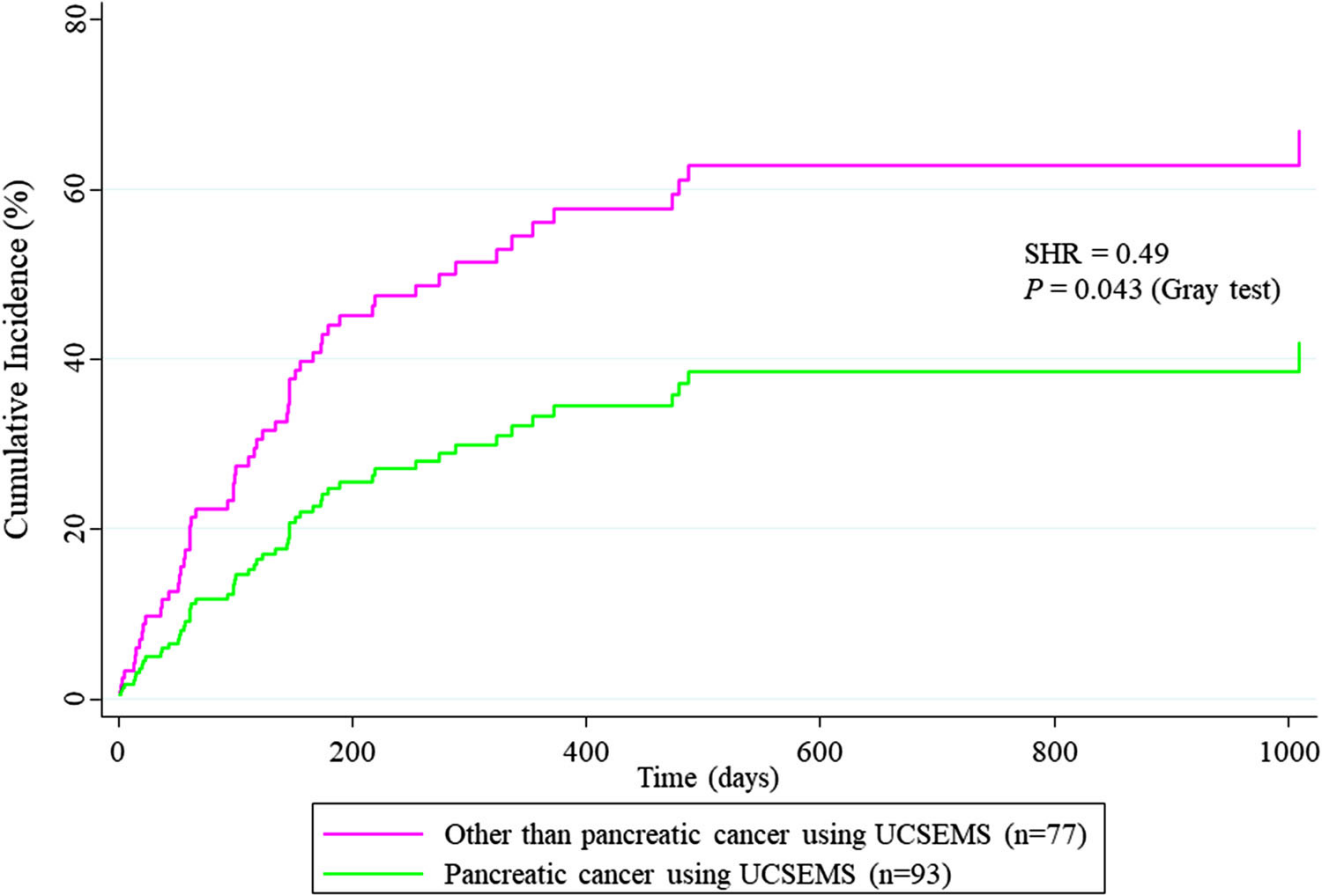
Comparison of time to recurrent biliary obstruction using propensity score‐adjusted competing‐risks regression between pancreatic cancer using uncovered self‐expandable metal stents and others. In the competing‐risks model for recurrent biliary obstruction rates, mortality was assigned as the competing risk. All variables listed in Table 1 were adjusted using propensity score analysis.

TRBO was estimated using the Kaplan–Meier analysis. Patient death was treated as a censor, and differences were evaluated using the log‐rank test for the Kaplan–Meier analysis (Figure 2a). The Fine and Gray model, based on a subdistribution hazard (SHR) model, was used for the competing‐risks regression (CRR).^4^, ^26^, ^28^ To include death in this model's informative censoring for potential RBO, death without RBO was treated as a competing risk. In this situation, differences were evaluated using the Gray test. TRBO‐associated factors were extracted using a multivariate CRR analysis to reduce confounders arising from background factors.^4^, ^26^, ^28^ In multivariate CRR, independent variables with *p*‐values < 0.15 in univariate CRR and type of SEMS were adopted. To adjust for differences in baseline‐related covariates as independent variables, CRR adjustment by propensity score analysis was employed to verify the robustness of the significance obtained from a multivariate CRR analysis.^29^, ^30^ The covariates used in the propensity score analysis were employed from all variables listed in Table 1. Finally, after the propensity score adjustment, SHRs were compared (Figures 2b and 3).

**TABLE 1:** Characteristics of patients with either fully covered or uncovered self‐expandable metallic stents (*n* = 180)

	**FCSEMS (*n* = 66)**	**UCSEMS (*n* = 114)**	***p‐*value**
Mean (range) age (years)	77.0 (36–98)	72.6 (37–93)	0.011^a^
No. of men (%)	35 (55.0)	70 (61.4)	0.27^b^
BMI, median (IQR)	20 (19–23)	20 (17–22)	0.18^a^
Acute cholangitis, *n* (%)	22 (33.3)	55 (48.3)	0.1^b^
Total bilirubin, mg/dl, median (IQR)	1.9 (1.0–4.2)	1.7 (0.7–4.6)	0.36^a^
Serum albumin, g/dl, median (IQR)	3.0 (2.3–3.4)	3.2 (2.7–3.5)	0.027^a^
CA19–9, U/ml, median (IQR)	248 (75–1675)	502 (112–1835)	0.28^a^
Primary malignancy, *n* (%)			
Pancreatic cancer	46 (69.7)	78 (68.4)	0.86^b^
Distal extrahepatic bile duct cancer	13 (19.7)	14 (12.3)	0.18^b^
Ampullary cancer	2 (3.0)	5 (4.4)	1.00^c^
Other cancer^d^	5 (7.5)	17 (14.9)	0.17^c^
Duodenal obstruction or invasion^e^	18 (27.3)	32 (28.1)	0.91^b^
Duodenal stent placement	8 (12.1)	13 (11.4)	0.89^b^
Gastrojejunal bypass	1 (1.5)	1 (0.9)	1.00^c^
UICC Stage, *n* (%)			
0/I/II	16 (24.2)	29 (2.54)	0.94^b^
III/IV	50 (75.8)	85 (75.2)	–
Chemotherapy after stent placement, *n* (%)	23 (34.9)	57 (50.0)	0.049^b^
Gemcitabine and TS‐1	5 (7.6)	14 (12.3)	0.45^c^
Gemcitabine and nab‐paclitaxel	6 (9.1)	11 (9.7)	0.90^b^
Gemcitabine	2 (3.0)	9 (7.9)	0.33^c^
TS‐1	4 (6.1)	7 (6.1)	1.00^c^
Other chemotherapy^f^	6 (26.1)	16 (28.1)	1.00^c^
Diameter of SEMS, *n* (%)			
10 mm	38 (57.6)	104 (92.0)	<0.001^b^
8 mm	22 (33.3)	10 (8.9)	<0.001^b^
6 mm	6 (9.1)	0 (0)	0.005^c^

^a^ Mann–Whitney test.

^b^ Chi‐square test.

^c^ Fisher's exact test.

^d^ Gallbladder cancer (*n* = 6), colon cancer (*n* = 6), gastric cancer (*n* = 5), breast cancer (*n* = 1), intrahepatic cholangiocarcinoma (*n* = 1), intraductal papillary mucinous carcinoma (*n* = 1), lung cancer (*n* = 1), ovarian cancer (*n* = 1).

^e^ Cases of endoscopic scope impassable due to obstruction of the upper digestive tract were excluded (see Figure 1), but scope‐passable stenosis with or without duodenal stent placement or gastrojejunal bypass was included.

^f^ Gemcitabine and cisplatin (*n* = 7), combination chemotherapy with fluorouracil, leucovorin, irinotecan, and oxaliplatin (FOLFIRINOX) (*n* = 6), nanoliposomal irinotecan and 5‐fluorouracil/ leucovorin (*n* = 1), capecitabine (*n* = 1), atezolizumab (*n* = 1), bevacizumab and paclitaxel (*n* = 1), nab‐paclitaxel (*n* = 1), nivolumab (*n* = 1), ramucirumab and paclitaxel (*n* = 1), bevacizumab and FOLFIRINOX (*n* = 1), erlotinib and gemcitabine (*n* = 1).

Abbreviations: BMI, body mass index; CA19‐9, carbohydrate antigen19‐9; FCSEMS, fully covered self‐expandable metallic stent; IQR, interquartile range; nab, nanoparticle albumin‐bound; UCSEMS, uncovered self‐expandable metallic stent.

Two‐sided *p*‐values <0.05 were considered statistically significant. All analyses were performed using Stata version 15 (StataCorp LP; College Station, TX, USA).

## RESULTS

### Patients’ characteristics

A total of 180 patients were included in this study. Of these, 66 received FCSEMS and 114 received UCSEMS. Detailed patient characteristics are presented in Table 1. There were no significant between‐group differences in sex, body mass index, presence of acute cholangitis, serum total bilirubin, serum carbohydrate antigen 19–9, primary malignancy, duodenal obstruction or invasion, or UICC stage. However, patients in the FCSEMS group were significantly older than those in the UCSEMS group (*p* = 0.011). Serum albumin levels were significantly lower in the FCSEMS group (*p* = 0.027). Although overall chemotherapy after stent placement was performed more frequently in the UCSEMs group than that in the FCSEMs group (*p* = 0.049), the four most frequently used chemotherapy regimens, that is, “gemcitabine and TS‐1,” “gemcitabine and nab‐paclitaxel,” “gemcitabine,” and “TS‐1,” showed no significant difference between the two groups. Ten‐millimeter stents were used significantly more often in the UCSEMS group (*p* < 0.001). However, 8‐ and 6‐mm stents were used significantly more in the FCSEMS than in the UCSEMS group (*p* < 0.001). Detailed information on SEMSs used in the two groups is presented in Table 2.

**TABLE 2:** Detailed information pertaining to self‐expandable metallic stents used in the two groups

**Stent type**	**FCSEMS (*n* = 66)**	**UCSEMS (*n* = 114)**
WallFlex Biliary RX Stent	34 (51.5)	71 (62.3)
HANAROSTENT	23 (34.8)	‒
X‐Suit NIR	6 (9.1)	‒
Niti‐S ComVi	3 (4.5)	‒
Niti‐S	‒	41 (36.0)
Zilver	‒	2 (1.8)

Abbreviations: FCSEMS, fully covered self‐expandable metallic stent; UCSEMS, uncovered self‐expandable metallic stent.

Pancreatic ductal carcinoma (PDAC) was the most common primary malignancy in 68.9% (*n* = 124) of the study population. Distal extrahepatic bile duct cancer and ampullary cancer were present in 15.0% (*n* = 27) and 3.9% (*n* = 7) patients, respectively. Other primary cancers included gallbladder cancer (*n* = 6), colon cancer (*n* = 6), gastric cancer (*n* = 5), breast cancer (*n* = 1), intrahepatic cholangiocarcinoma (*n* = 1), intraductal papillary mucinous carcinoma *(n* = 1), lung cancer (*n* = 1), and ovarian cancer (*n* = 1).

### Comparison of the outcomes between the FCSEMS and UCSEMS groups

A comparative analysis of the between‐group outcomes is presented in Table 3. Technical success was achieved in all cases, and the clinical success rate was 91.8% and 92.9% in the FCSEMS and UCSEMS groups, respectively. There were no significant between‐group differences in technical and clinical success rates.

**TABLE 3:** Characteristics of endoscopic biliary drainage and outcomes between fully covered and uncovered self‐expandable metallic stents

	**FCSEMS (*n* = 66)**	**UCSEMS (*n* = 114)**	***p‐*value**
Technical success rates, % (95% CI)	100 (94.6–100)	100 (96.8–100)	1.00^a^
Functional success rates, % (95% CI)	91.8 (81.8–97.3)	92.9 (86.5–96.9)	0.77^a^
Time to functional success, Mean ± SD, days	5.2 ± 4.7	4.3 ± 3.4	0.69^b^
RBO, *n* (%)	20 (30.3)	43 (37.7)	0.34^c^
Median TRBO (IQR), days	275 (147–373)	255 (100–1010)	0.67^d^
Non‐obstruction rates, % (95% CI)			
at 3 months	82.4 (68.6–90.6)	80.2 (70.3–87.1)	0.72^c^
at 6 months	55.6 (36.1–71.3)	55.4 (42.9–66.2)	0.97^c^
at 12 months	24.7 (7.1–47.8)	41.1 (26.8–54.9)	0.026^c^
Cause of RBO, *n* (%)			
Tumor ingrowth	0 (0)	30 (26.3)	<0.001^a^
Tumor overgrowth	6 (9.2)	5 (4.4)	0.22^a^
Symptomatic migration in distal	7 (10.6)	1 (0.9)	0.004^a^
Sludge/food impaction	3 (4.6)	2 (1.8)	0.36^a^
Hemobilia	0 (0)	3 (2.6)	0.30^a^
Kinking of bile duct with SEMS	1 (1.5)	1 (0.9)	1.00^a^
Sludge formation	1 (1.5)	1 (0.9)	1.00^a^
Unknown	2 (3.0)	0 (0)	0.13^a^

Abbreviations: CI, confidence interval; FCSEMS, fully covered self‐expandable metallic stent; IQR, interquartile range; RBO, recurrent biliary obstruction; SD, standard deviation; TRBO, time to recurrent biliary obstruction; UCSEMS, uncovered self‐expandable metallic stent.

^a^ Fisher's exact test.

^b^ Mann–Whitney test.

^c^ Chi‐square test.

^d^ Log‐rank test.

The causes of RBO are listed in Table 3. RBO occurred in 20 (30.3%) and 43 (37.7%) patients in the FCSEMS and UCSEMS groups, respectively. The primary cause of RBO in the UCSEMS group was tumor ingrowth (30/43), although no instances of tumor ingrowth occurred in the FCSEMS group (*p* < 0.001). The most frequent cause of RBO in the FCSEMS group was distal stent migration (7/20), which occurred significantly more frequently than in the UCSEMS group (*p* = 0.004). To investigate factors associated with FCSEMS migration, we compared the backgrounds of seven cases with RBO caused by migration and 13 cases with RBO caused by factors other than migration within the FCSEMS group (Table 4). Chemotherapy was administered more frequently after FCSEMS placement in RBO owing to migration than in RBO owing to other causes (*p* = 0.015). All seven cases of RBO owing to migration received chemotherapy after FCSEMS placement, whereas in 13 cases of RBO due to reasons other than migration, only five (38.5%) received chemotherapy after FCSEMS placement (Table 4). In patients who received UCSEMS with chemotherapy, 18/57 (31.6%) had RBO due to ingrowth. The frequency was similar to 12/57 (21.1%) in patients who received UCSEMS without chemotherapy (no Table).

**TABLE 4:** Comparison of background distribution within FCSEMS and RBO group with and without migration in distal (*n* = 20)

	**RBO owing to migration (*n* = 7)**	**RBO other than migration (*n* = 13)**	***p‐*value**
Mean (range) age (years)	68.4 (43–83)	73.9 (52–95)	0.43^a^
No. males (%)	3 (42.9)	5 (38.5)	1.00^b^
BMI, mean (±SD)	21.1 (±3.0)	20.9 (±3.9)	0.63^a^
Acute cholangitis, *n* (%)	3 (42.9)	6 (46.2)	1.00^b^
Total bilirubin, mg/dl, mean (±SD)	1.8 (±1.5)	2.5 (±2.0)	0.45^a^
Serum albumin, g/dl, mean (±SD)	3.5 (±0.4)	3.2 (±0.7)	0.23^a^
CA19‐9, U/ml, mean (±SD)	2486.7 (±3753.9)	4340.3 (±9256.5)	0.84^a^
Final diagnosis and Indications of SEMS, *n* (%)			
Pancreatic cancer	7 (100)	9 (69.2)	0.25^c^
Distal extrahepatic bile duct cancer	0 (0)	2 (15.4)	0.52^b^
Other cancer^d^	0 (0)	2 (15.4)	0.52^b^
UICC Stage, *n* (%)			
0/I/II	1 (14.3)	4 (30.8)	0.61^b^
III/IV	6 (85.7)	9 (69.2)	–
Chemotherapy after stent placement, *n* (%)	7 (100)	5 (38.5)	0.015^b^
Diameter of SEMS, *n* (%)			
10 mm	4 (57.1)	10 (76.9)	0.61^b^
8 mm	2 (28.6)	2 (15.4)	0.59^b^
6 mm	1 (14.3)	1 (7.7)	1.00^b^
Technical success rates, % (95% CI)	100 (59.0–100)	100 (75.3–100)	1.00^b^
Functional success rates, % (95% CI)	85.7 (42.1–99.6)	100 (69.2–100)	0.41^b^
Median TRBO, (IQR), days	147 (3–275)	63 (21–152)	0.81^e^
Non‐obstruction rates, % (95% CI)			
at 3 months	57.1 (17.2–83.7)	38.5 (14.1–62.8)	0.43^c^
at 6 months	28.6 (4.1–61.2)	23.1 (5.6–47.5)	0.79^c^
at 12 months	0 (0)	7.7 (0.5–29.2)	0.45^b^

Abbreviations: BMI, body mass index; CA19‐9, carbohydrate antigen19‐9; CI, confidence interval; FCSEMS, fully covered self‐expandable metallic stent; IQR, interquartile range; RBO, recurrent biliary obstruction; SD, standard deviation; TRBO, time to recurrent biliary obstruction.

^a^ Mann–Whitney test.

^b^ Fisher's exact test.

^c^ Chi‐square test.

^d^ Gallbladder cancer (*n* = 1), colon cancer (*n* = 1).

^e^ Log‐rank test.

The median TRBO estimated using the Kaplan–Meier method was 275 and 255 days in the FCSEMS and UCSEMS groups, respectively, indicating no significant between‐group difference (*p* = 0.67, Log‐rank test; Figure 2a and Table 3). Non‐obstruction rates at 3, 6, and 12 months were 82.4%, 55.6%, and 24.7% in the FCSEMS group, and 80.2%, 55.4%, and 41.1% in the UCSEMS group, respectively.

In propensity score‐adjusted CRR, there was no significant between‐group difference in TRBO (SHR = 1.02, *p* = 0.96, Gray test; Figure 2b).

### Complications other than RBO

The complications other than RBO are listed in Table 5. The incidence of all complications in the FCSEMS and UCSEMS groups was 21.2% (14/66) and 8.8% (10/114), respectively; the FCSEMS group experienced significantly more complications (*p* = 0.023). Complications included pancreatitis (*n* = 6), cholecystitis (*n* = 4), non‐occlusion cholangitis (*n* = 2), and liver abscess (*n* = 2) in the FCSEMS group and pancreatitis (*n* = 4), cholecystitis (*n* = 1), non‐occlusion cholangitis (*n* = 4), and liver abscess (*n* = 1) in the UCSEMS group. Although there was no significant difference between the groups for each individual complication, pancreatitis and cholecystitis tended to be more frequent in the FCSEMS group than in the UCSEMS group. Three of the six patients with pancreatitis in the FCSEMS group and one of the four patients with pancreatitis in the UCSEMS group were DMBO patients derived from PDAC. Involvement in the orifice of the cystic duct was observed in two of four patients with cholecystitis in the FCSEMS group and one in one in the UCSEMS group.

**TABLE 5:** Complications other than recurrent biliary obstruction

	**FCSEMS (*n* = 66)**	**UCSEMS (*n* = 114)**	*p‐*value^a^
Pancreatitis	6 (9.1)	4 (3.5)	0.17
Mild	6 (9.1)	3 (2.6)	–
Severe	0	1 (0.9)	–
In patients with pancreatic cancer	3 (4.6)	1 (0.9)	–
Cholecystitis	4 (6.1)	1 (0.9)	0.06
With OCD involvement	2 (3.0)	1 (0.9)	–
Non‐occlusion cholangitis	2 (3.0)	4 (3.5)	1.00
Liver abscess	2 (3.0)	1 (0.9)	0.56
Total	14 (21.2)	10 (8.8)	0.023

Abbreviations: DMBO, distal malignant biliary obstruction; FCSEMS, fully covered self‐expandable metallic stent; OCD, the orifice of the cystic duct; UCSEMS, uncovered self‐expandable metallic stent.

*Note*: Numbers are shown in *n* (%).

^a^Fisher's exact test.

### Factors associated with TRBO

The univariate and multivariate CRR for the factors associated with TRBO are shown in Table 6. Univariate analysis revealed a significantly longer TRBO with PDAC (SHR = 0.41, 95% confidence interval [CI] 0.20–0.84, *p* = 0.015) and PDAC using UCSEMS (SHR = 0.56, 95% CI 0.34–0.92, *p* = 0.004). Although all cases of RBO arising from migration received chemotherapy after FCSEMS placement (Table 4), chemotherapy was not identified as a TRBO‐related factor (SHR = 1.16, 95% CI 0.71–1.88, *p* = 0.56; Table 6).

**TABLE 6:** Factors associated with time to recurrent biliary obstruction using competing‐risks regression (*n* = 180)

**Independent variables**		**Univariable analysis** **^a^**		**Multivariate analysis** **^a^**	
**(RBO = 63, Compete = 54, Censored = 63)**	***n***	**SHR (95% CI)**	***p‐*value** **^b^**	**SHR (95% CI)**	***p‐*value** **^b^**
Type of SEMS					
UCSEMS	114	Reference	–	Reference	–
FCSEMS	66	0.97 (0.59–1.62)	0.92	0.75 (0.40–1.40)	0.37
Diameter of SEMS					
FCSEMS or UCSEMS 10 mm	142	Reference	–		
FCSEMS or UCSEMS 8 mm	32	1.08 (0.56–2.09)	0.81		
FCSEMS 6 mm	6	2.59 (0.38–17.52)	0.33		
Age > 74	102	0.63 (0.39–1.03)	0.06	0.70 (0.40–1.22)	0.20
Men	105	1.24 (0.75–2.05)	0.4		
BMI > 20	73	1.13 (0.70–1.83)	0.63		
Anti_PLT	24	1.00 (0.45–2.24)	1.00		
Anti_Coag	10	2.01 (0.80–5.09)	0.14	1.99 (0.81–4.90)	0.14
CA19‐9 > 342 ng/ml	86	0.70 (0.42–1.14)	0.15	0.73 (0.44–1.21)	0.22
Alb > 3.0 g/dl	93	1.46 (0.89–2.40)	0.14	1.45 (0.82–2.55)	0.20
T‐Bil > 3.4 mg/dl	56	0.84 (0.49–1.45)	0.53		
Acute cholangitis	103	0.73 (0.45–1.19)	0.20		
Length of biliary stricture > 26 mm	77	1.08 (0.55–2.12)	0.82		
Type of major papilla					
Post plastic stenting	97	Reference	–		
Naïve papilla	25	0.52 (0.23–1.23)	0.14	0.55 (0.21–1.39)	0.20
Post EST	58	0.78 (0.46–1.32)	0.35		
Chemotherapy after SEMS placement	80	1.16 (0.71–1.88)	0.56		
Type of SEMS and chemotherapy					
UCSEMS without chemotherapy	57	Reference	–		
UCSEMS with chemotherapy	57	0.83 (0.46–1.51)	0.55		
FCSEMS without chemotherapy	43	0.58 (0.26–1.32)	0.20		
FCSEMS with chemotherapy	23	1.29 (0.66–2.50)	0.45		
Primary malignancy					
Other malignant biliary stricture^c^	22	Reference	–		
Ampullary cancer	7	0.72 (0.13–3.99)	0.71		
Distal extrahepatic bile duct cancer	27	0.78 (0.36–1.67)	0.52		
Pancreatic cancer	124	0.41 (0.20–0.84)	0.015		
Pancreatic cancer using FCSEMS	46	0.95 (0.55–1.64)	0.86		
Pancreatic cancer using UCSEMS	78	0.56 (0.34–0.92)	0.004	0.51 (0.28–0.92)	0.03
Duodenal obstruction or invasion^d^	50	1.11 (0.67–1.84)			
Stage III/IV	135	0.98 (0.59–1.62)	0.94		

^a^ Fine and Gray model.

^b^ Gray test.

^c^ Gallbladder cancer (*n* = 6), colon cancer (*n* = 6), gastric cancer (*n* = 5), breast cancer (*n* = 1), intrahepatic cholangiocarcinoma (*n* = 1), intraductal papillary mucinous carcinoma (*n* = 1), lung cancer (*n* = 1), ovarian cancer (*n* = 1).

^d^ Cases of endoscopic scope impassable due to obstruction of the upper digestive tract were excluded (See Figure 1), but scope‐passable stenosis with or without duodenal stent placement or gastrojejunal bypass was included.

Abbreviations: Anti_Coag, anticoagulant; Anti_PLT, antiplatelet agent; BMI, body mass index; CA19‐9, carbohydrate antigen19‐9; CI, confidence interval; FCSEMS, fully covered self‐expandable metallic stent; RBO, recurrent biliary obstruction; SHR, subdistribution hazard ratio; UCSEMS, uncovered self‐expandable metallic stent.

In multivariate CRR analysis, “PDAC using UCSEMS” was the only independent factor for TRBO (SHR = 0.51, 95% CI 0.28–0.92, *p* = 0.03; Table 6). The propensity score‐adjusted CRR also showed that “PDAC using UCSEMS” was a significant independent factor for long TRBO (SHR = 0.49, 95% CI 0.25–0.98, *p* = 0.043).

### TRBO of FCSEMS and UCSEMS within the non‐chemotherapy group (terminal care in all DMBO)

**FIGURE 4:**
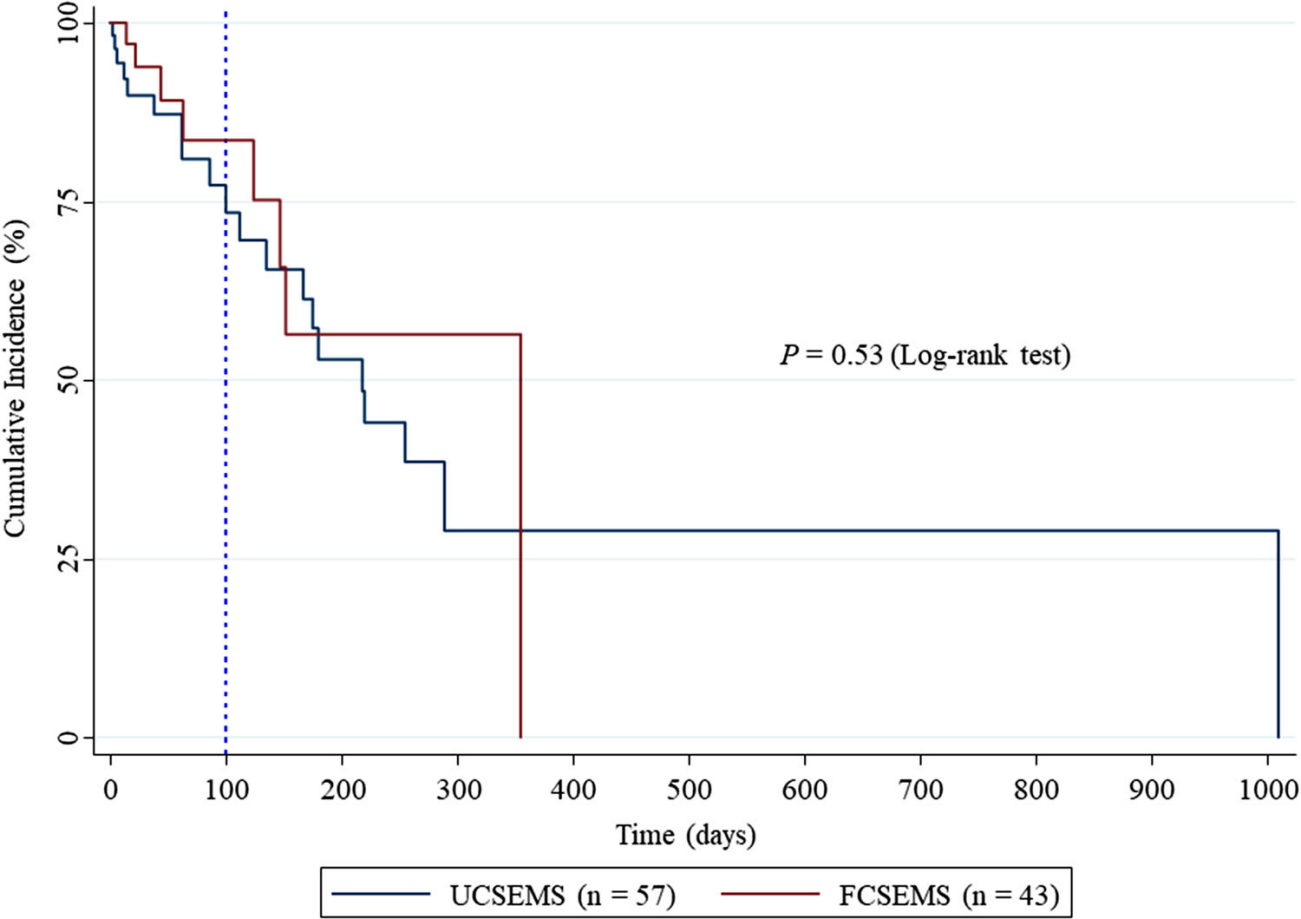
Kaplan–Meier curves compare time to recurrent biliary obstruction in patients with distal malignant biliary obstruction treated without chemotherapy between uncovered self‐expandable metal stent and fully covered self‐expandable metal stent placement. The vertical dotted line shows day 100, the first quartile of time to recurrent biliary obstruction.

No significant difference between the FCSEMS and UCSEMS was observed for patients receiving terminal care only (i.e., non‐chemotherapy group). In addition, the first quartile of TRBO of UCSEMS was 100 days (Figure 4). In other words, more than 75% of best supportive care cases did not develop RBO after UCSEMS placement within 100 days (Figure 4).

## DISCUSSION

FCSEMSs and UCSEMSs provide comparable TRBO in DMBO in patients who are not surgical candidates. Although this was a retrospective study, we tried to approximate a randomized controlled trial using propensity score‐adjusted CRR analysis. Our results resemble those of a recent meta‐analysis.^21^ Moreover, we found that “PDAC using UCSEMS” was a significant independent factor for long TRBO.

The FCSEMS group had more frequent complications than the UCSEMS group. This finding is unlike those of prior meta‐analyses that found similar complication rates for CSEMSs and UCSEMSs.^18^, ^19^, ^20^ One of the reasons for the abovementioned difference might be that the covered Wallflex Biliary RX Stent with a high axial force was frequently used in this study because SEMSs with high axial force increase the risk of pancreatitis and cholecystitis.^31^, ^32^

As previously reported,^20^ we also found that the leading causes of RBO were ingrowth for the UCSEMS group and distal migration for the FCSEMS group. In the FCSEMS group, all seven cases with distal migration underwent chemotherapy after SEMS placement. Nakai et al. reported that anticancer treatment was a risk factor for RBO in CSEMS for DMBO.^33^ On the contrary, Kang et al. reported that, compared with patients who received supportive care only, those who underwent chemotherapy after SEMS insertion for DMBO had better stent patency.^34^ Although this result is inconsistent with Nakai et al., 78% of SEMSs used in this study were UCSEMs. Therefore, chemotherapy seems to promote distal migration in FCSEMS and suppress ingrowth in UCSEMS. In our study, chemotherapy was not extracted as a factor related to TRBO in both FCSEMS and UCSEMS for DMBO cases (Table 6). However, migration risk should be carefully monitored in patients undergoing chemotherapy with FCSEMS placement because the proportion of RBO arising from migration was significantly higher than that of other RBOs (Table 4).

In addition to its other advantages, FCSEMS reinsertion after removal is often easier than UCSEMS placement at the time of reintervention using the stent‐in‐stent technique. In other words, FCSEMSs can be easily removed and replaced when the length and the location of the stenosis changes with cancer progression. Therefore, if TRBO and complications are equivalent, FCSEMS should be selected in patients with an estimated prognosis exceeding TRBO and where future reintervention is expected.

In contrast, for patients where the prognosis is poor and reintervention is likely not required, the benefits of using FCSEMSs are small; UCSEMSs may be a good option for these patients for the following three reasons: 1) propensity score‐adjusted CRR showed that FCSEMSs and UCSEMSs provide comparable TRBO in DMBO as a whole (Figure 2B), 2) complications in the UCSEMS group were fewer and less frequent than those in the FCSEMS group, and 3) the first quartile of TRBO in patients with DMBO treated without chemotherapy after UCSEMS placement was 100 days (Figure 4). Moreover, although PS is recommended for patients with a prognosis of less than 3 months,^35^ we previously reported that the first quartile of TRBO in patients with DMBO after PS placement was approximately 1 month.^4^ The results of the present study and our previous report^4^ suggest that UCSEMSs may be a good option for patients receiving supportive care having a prognosis of 30–100 days.

This study has several limitations. First, our study cohort was relatively small, and data were collected retrospectively from only two centers. Second, using multiple types of SEMSs for both FCSEMS and UCSEMS might have affected the results. Finally, although propensity score‐adjusted CRR analyses were used, unobserved selection biases and potential confounding factors may remain. Therefore, a well‐powered, multicenter, prospective study should be completed to confirm our findings.

In conclusion, UCSEMSs are a good option for patients with DMBO arising from PDAC and for patients with any DMBO receiving palliative care only in cases where undesirable complications from SEMS develop and reinterventions are unlikely.

## CONFLICT OF INTEREST

The author Kazuki Sumiyama is DEIC of DEN Open. The rest of the authors declare that they have no conflict of interest.

## FUNDING INFORMATION

None.
